# Editorial: Advances on targeted nanomedicines for atherosclerosis

**DOI:** 10.3389/fphar.2022.1053938

**Published:** 2022-10-14

**Authors:** Qinggong Han, Hongliang He, Yu Zhang

**Affiliations:** State Key Laboratory of Bioelectronics, Jiangsu Key Laboratory for Biomaterials and Devices, School of Biological Sciences and Medical Engineering, Southeast University, Nanjing, China

**Keywords:** nanomedicine, atheroscelrosis, macrophage, inflammation, imaging

## Abstract

The importance of nanomedicines for atherosclerosis.
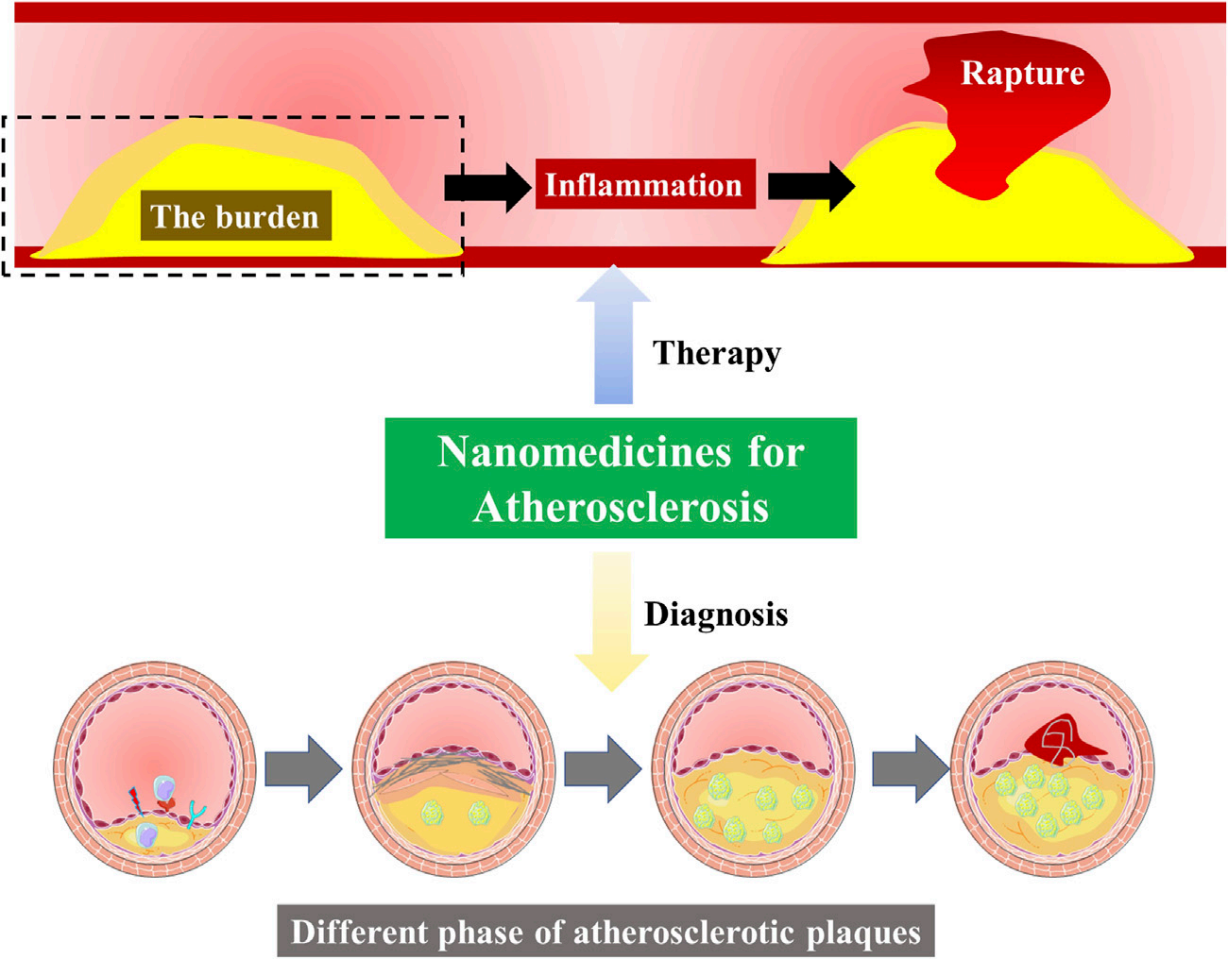

Atherosclerosis is the primary pathological foundation that underlies acute cardiovascular and cerebrovascular events, including heart attack and stroke. The iconic feature of atherosclerosis is the plaques formed within the arterial wall, due to the accumulation of cholesterol ester-laden macrophages, inflammation-induced cell apoptosis, and the formation of a necrotic core. The main pathogenesis of atherosclerosis involves lipid accumulation-induced inflammation. First, the excessive intake of modified LDL by macrophages in the arterial intima is the basis for lipid deposition, thus leading to inflammation and plaque growth. As the disease progresses, excess lipid accumulation induces macrophage apoptosis, and the accumulation of apoptotic cells induces secondary necrosis (the emergence of necrotic cores), thus promoting inflammation, plaque instability, and acute thrombosis (Bäck et al., 2019).

Although the current clinical options (lipid-lowering drugs and anti-inflammatory drugs) prevent the progression of plaque and reduce the incidence of acute events, the overall clinical efficacy is still not satisfactory. Even after the drug treatment achieves lipid control, the existing plaques in the blood vessels will still bring potential cardiovascular risk to patients. The incidence of cardiovascular disease keeps increasing year by year. Dissecting the reasons behind this severe clinical situation, besides the high-fat diet and unhealthy lifestyle as important external stimuli, the current clinical inadequacy of antiatherogenic therapeutics is the key internal cause of poor prevention and treatment of cardiovascular disease (Waterbury et al., 2020; Roy et al., 2021).

First, systemic exposure and drug-drug interactions remain a concern for nearly all conventional antiatherogenic therapies, leading to low bioavailability and poor targeting *in vivo*. Therefore, the search for an efficient drug delivery approach is highly imminent. In recent years, nanomedicines promise to advance strategies to treat atherosclerosis. It can effectively improve the therapeutic effect, improve biological distribution, and reduce drug toxicity. Second, no drug can relieve the existing plaque burden. The lipid deposition and apoptotic cells continue to induce inflammatory responses, reduce plaque stability, and increase the risk of recurrence and acute events in patients. Therefore, to effectively stabilize plaque, it is necessary to develop targeted nanomedicine that can reduce the burden of plaque (Soehnlein and Libby, 2021).

Recently, amounting evidence demonstrates that macrophages play a critical role in the regulation of inflammation. However, abnormal cell metabolisms under pathological conditions impair the innate inflammatory regulation function of macrophages, leading to the persistence of inflammation. So, restoring the function of macrophages to regulate inflammation *via* nanomedicine is expected to become a safer and more effective inflammation-resolving strategy, which avoids systemic immunosuppression induced by systemic anti-inflammatory drugs that occurred in the CANTOS trial and the COLCOT trial (Ridker et al., 2017; Chen et al., 2021).

In addition to the nanomedicine for preventing atherosclerosis, early detection of atherosclerotic plaque provides rapid treatment for patients while monitoring plaque size and composition before and during treatment to assess the efficacy and know appropriate medication regimen adjustments, further guiding the individualized choice of drug therapy to maximize patient benefits (Perkins et al., 2019).

In the Research Topic, Hossaini Nasr et al. and Zhang et al. contributed two insightful reviews, respectively. Those two reviews are highly complementary to each other. Hossaini Nasr et al. mainly focused on the various strategies (using natural lipoproteins, cells, biomacromolecules, etc.) for active targeting of atherosclerotic plaques, including macrophages, adhesion molecules, and extracellular matrix. Zhang et al. comprehensively summarized the advances in the development of Nanomedicines–based imaging techniques for precise assessment of the degree of atherosclerotic plaque. In addition, both reviews pointed out the future directions for this field, the remaining challenges to overcome, and the rational design principles for atheroprotective nanocarriers from distinct perspectives. We believe that they will provide valuable information for researchers in this field.

Targeting the critical elements involved in the development of atherosclerosis would be a promising option, among them, the injured vascular endothelium plays important role in the initiation and progression of atherosclerosis. High-density lipoproteins (HDLs) play crucial roles in regulating inflammation responses and endothelial functions. HDLs have been reported to reduce the expression of adhesion molecules, alleviate intracellular oxidative stress, prevent endothelial cell apoptosis, and inhibit the secretion of proinflammatory cytokines on activated endothelial cells. In this Research Topic, Yu et al. developed a novel inflamed endothelium-targeting synthetic HDLs (sHDLs) *via* vascular cell adhesion protein 1 specific VHPK peptide. The active inflamed endothelium targeting of VHPK-sHDLs was confirmed on TNF-α injured endothelial cells. VHPK-sHDLs exerted strong anti-inflammatory effects indicated by the reduction of proinflammatory cytokines and leukocyte adhesion to activated endothelium. Moreover, they demonstrated that VHPK-sHDLs strongly bound to inflamed vessels *in vivo* and alleviated LPS-induced lung inflammation in a diseased mouse model. The VHPK-sHDLs may be further optimized and adapted to resolve endothelial inflammation in various inflammatory disease settings.

Experimental and clinical evidence suggests that the immune system is involved at all stages of atherosclerosis. In the original research, Yang et al. uncovered the roles of several immune-associated genes (TNFSF13B, CCL5, CCL19, ITGAL, CD14, GZMB, and BTK) during the progression of atherosclerotic plaques. Higher immune cell infiltrations and immune checkpoint expressions were observed in advanced atherosclerotic plaques than in early atherosclerotic plaques and were positively associated with those characteristic genes. In addition, several small molecular compounds were targeted based on those characteristic genes. In the clinic, patients could benefit from the characteristic gene-based nomogram. Their findings could assist the design of more accurate atheroprotective immunotherapy.

Overall, the collections of research articles and reviews on this Research Topic will encourage us to explore more promising therapeutic targets and facilitate the critical evaluation of novel therapeutics against atherosclerosis. In summary, with the first successful commercial approval of Inclisiran (a PCSK9-targeted double-stranded small interfering RNA that can inhibit the PCSK9 mRNA, thus reducing the synthesis of the PCSK9 protein, thereby reducing plasma LDL-C levels) and the promising clinical efficacy of a novel lipoprotein-based therapy CSL112 in Phase 3 (The AEGIS-II trial), the future of nanomedicine for atherosclerosis shall be even more bright and promising.
